# Does Existential Flexibility Associate With Individuals’ Acceptance of Inequality? A Study Relating Existential Questing to Values and to Prejudice

**DOI:** 10.5964/ejop.9999

**Published:** 2023-11-30

**Authors:** Anna Miglietta, Marco Rizzo, Silvia Testa, Silvia Gattino

**Affiliations:** 1 Department of Psychology, University of Turin, Turin, Italy; 2 Department of Humanities and Social Sciences, University of Aosta Valley, Aosta, Italy; Victoria University of Wellington, Wellington, New Zealand

**Keywords:** existential quest, values, universalism, conservation, generalized prejudice

## Abstract

This study investigated whether existential quest, a relatively new construct defining individual willingness to reflect on existential issues such as the meaning of life and death, was negatively associated with generalized prejudice through the mediation with personal values of universalism and conservation (conformity, security, and tradition). A structural equation model was performed on a convenience sample of 1136 Italian adults. Results confirmed a negative indirect relationship with generalized prejudice mediated by universalism. Findings support the argument that engagement with existential issues is associated with the value of universalism, which in turn is associated with lower levels of generalized prejudice. The present study contributes to the scholarly literature to explain the concept of existential quest.

Contemporary times are characterized by increasing negative attitudes in Western societies towards diversity and disadvantaged social groups such as migrants (Esses, 2021; Servidio et al., 2021), the LGBTQI+ community (Earle et al., 2021; Morrison & Morrison, 2011; Worthen et al., 2017), or Muslim people (Adelman & Verkuyten, 2020; Strabac & Listhaug, 2008). The rise of prejudice, racism, and discrimination is a clear indicator of the difficulties that Western societies face in integrating diverse individuals and groups, as shown by the latest report of the European Union Agency for Fundamental Rights (FRA, 2022). Derogatory attitudes are not only an obstacle to integration and social cohesion, but also easily create a context of hatred and mistrust that, under certain social conditions, can lead to harmful behaviors such as discrimination, exclusion, or, even worse, brutality and violence.

Decades of research in social psychology have consistently shown that prejudice against minorities is related to various aspects. Two primary and complementary research traditions can be identified in these issues. One important tradition sees the basis of prejudice in structural and contextual factors that interact with intergroup dynamics. Sherif and colleagues’ (1961) realistic conflict theory, Tajfel’s CIC approach (social Categorization, social Identity, social Comparison; Tajfel 1982), and relative deprivation theory (Runciman, 1966) itself underscore the importance of considering aspects related to intergroup functional interdependence as possible explanations for intergroup prejudice and hostility. In addition, other research focuses more on psychological elements that explain why people think in terms of prejudice, as in the case of studies on intergroup threat theory (Aberson et al., 2021; Stephan et al., 2016). The present study follows this tradition, as it focuses on the role of the individual value system (Schwartz, 1992) in orienting attitudes toward other groups. As abstract and trans-situational goals, values drive people to interpret the social world and orient to different behaviors depending on what they consider “good” or “bad” (Piurko et al., 2011; Schwartz, 1992; Vecchione et al., 2012).

The importance of values in advocating derogatory attitudes toward minorities is a consistent research finding (Feather & McKee, 2008; Sagiv & Schwartz, 2022). A large body of research suggests a link between endorsing conservative values and holding negative intergroup attitudes such as stereotyping, prejudice, and intolerance, if not hostility, toward a variety of minority groups (e.g., Barni et al., 2020; Leong & Ward, 2006; Ponizovskiy, 2016). Interestingly, research has also found that conservatives who hold negative attitudes toward diversity are strongly motivated to perform psychological processes to reduce cognitive uncertainty, such as cognitive closure (Jost, Blount, et al., 2003; Jost, Glaser, et al., 2003). Indeed, studies have consistently shown that people with a high need for cognitive closure, a construct that implies individual close-mindedness (Webster & Kruglanski, 1994), are more likely to engage in readily available knowledge that helps them have a point of reference in uncertain situations (or a top-down tendency; Golec De Zavala et al., 2008). Moreover, empirical research confirmed the link between the need for cognitive closure and ethnic prejudice or negative outgroup attitudes (Baldner et al., 2019; Sibley & Duckitt, 2008). Finally, Jost, Blount, et al. (2003) and Jost, Glaser, et al. (2003) have shown that the higher the cognitive closure, the greater the possibility of supporting conservative values that tend to seek order and exclude diversity.

In the same studies, Jost, Blount, et al. (2003) and Jost, Glaser, et al. (2003) found evidence of a positive relationship between the need for cognition, the preference for values of universalism and the acceptance of diversity. The need for cognition is an epistemic need (Cacioppo & Petty, 1982) that refers to the individual's search for an answer to a cognitive effort and can be understood as the opposite of the need for cognitive closure. The relationship between universalism and acceptance of diversity is consistent with other research highlighting the positive relationship between values of self-transcendence and positive attitudes toward minorities (Davidov et al., 2020; Feather & McKee, 2008; Saroglou et al., 2009).

Within this framework, understanding the relationships among epistemic needs, values, and intergroup attitudes can shed light on the psychological characteristics of those who express exclusive versus inclusive attitudes toward minorities. The current research aims to understand whether epistemic needs other than the need for cognitive closure and the need for cognition may orient the individual relationship between values and prejudice toward minority groups. In this context, we investigate whether existential quest (EQ; Van Pachterbeke et al, 2012) represents a further psychological variable that can be included into the relationship between values and derogatory attitudes. EQ refers to an individual difference in the flexibility of one’s belief system regarding existential issues, i.e., the willingness to examine and change one’s beliefs regarding central and universal issues. We expected that a high quest orientation would drive people toward values of universalism which in turn would lead to more positive attitudes toward minority outgroups consistent with its defining goal: “understanding, appreciation, tolerance, and protection for the welfare of all people and for nature” (Schwartz, 2012, p. 7). This expectation was supported by recent findings (Arrowood et al., 2022) showing that individuals high in quest orientation show, among others, a more significant emphatic concern, perspective-taking, and openness (Ghorbani et al., 2007; Henningsgaard & Arnau, 2008), as well as increased tolerance and acceptance of those with culturally different ways of life (Van Tongeren et al., 2016).

Because of its relative novelty, the literature on the EQ construct is still sparse, and our secondary aim in the present study is to add empirical evidence to this concept. Nevertheless, some empirical studies have shown that EQ is negatively associated with the need for cognitive closure (Rizzo et al., 2019; Van Pachterbeke et al., 2012). This is consistent with EQ's focus on people's tendency to think about existential issues through an openness to change. A positive evaluation of doubt (Van Pachterbeke et al., 2012) corresponds to a quest attitude (Arrowood et al., 2022) that contrasts with the rigidity of thinking emphasized by the need for cognitive closure. In addition, recent research has shown that people who score low on EQ are likely to have negative attitudes toward religious people, suggesting an overall close-mindedness (Uzarevic et al., 2021). Given the negative associations between EQ and the need for cognitive closure, e.g., with derogatory attitudes toward minorities, we hypothesize that questioning existential issues may be related to a greater individual acceptance of diversity in society.

## Personal Values and Generalized Prejudice

Prejudice may be directed toward a wide range of disadvantaged groups. Nevertheless, as Allport (1954, p. 68) noted, “people who reject one out-group will tend to reject other out-groups. If a person is anti-Jewish, he is likely to be anti-Catholic, anti-Negro, anti any out-group”. The affective proximity of the different types of prejudice suggests that derogative attitudes toward outgroups are interrelated, beyond each group’s specific history and connotations (see Everett et al., 2019). A decade ago, Zick and colleagues (2008) proposed that different kinds of prejudice can be conceptually grouped under a syndrome labeled group-focused enmity (GFE). GFE “encompasses prejudices toward different groups that are, within a stable structure, substantially interrelated over a period of time even though the level of approval can vary across time, cultures, and individuals” (Zick et al., 2008, p. 364). GFE is powered by a core consisting of devaluing attitudes that are predicted in turn by support for an ideology of inequality (Küpper & Zick, 2014; Zick et al., 2008). As research has shown, ideologies of inequality serve the majority group interested in maintaining or enhancing its own status and, at the same time, serve to keep members of lower-status groups in their place (Sidanius & Pratto, 2004).

Validation studies proved the existence of GFE syndrome using cross-sectional and longitudinal survey data from Germany (Davidov et al., 2011; Zick et al., 2008) and a large cross-national study involving eight European countries (Zick et al., 2011). The results consistently showed the existence of a devaluing and inequality-supporting common core strictly related to social dominance orientation (Sidanius & Pratto, 2004) and right-wing authoritarianism (Altemeyer & Hunsberger, 1992). The predictive role of GFE for prejudicial attitudes toward a wide range of social targets, e.g., sexism, anti-Semitism, and ethnic prejudice, was confirmed by a recent study that also highlighted the relationships between GFE and individuals’ value priorities (Beierlein et al., 2016). This is in line with previous findings on the relationship between prejudicial attitudes and personal values, (e.g., Becker et al., 2012; Davidov & Meuleman, 2012; Kuntz et al., 2015; Miglietta et al., 2018). Specifically, Beierlein and colleagues (2016) found a positive contribution of the values of conservation (security, tradition, and conformity) to GFE and a negative contribution of the value of universalism. Similarly, Davidov and colleagues (2020) showed across Western and Eastern European countries that universalism was associated with a greater support of immigration than conformity and tradition, which revealed strong association with the rejection of immigration.

Interesting as it is, this relationship is not surprising. In fact, on the one hand, evidence from studies in political psychology highlights that the endorsement of values of conservation enhances a right-wing political orientation, supporting ideologies of inequality (Jost, Blount, et al., 2003; Jost, Glaser, et al., 2003), which are also at the core of GFE (Vecchione et al., 2012; cf. Datler et al., 2013). On the other hand, research shows that self-transcendence values positively relate to support for human and minority rights (Kuşdil & Şimşek, 2008), willingness to engage in contact with outgroup members (Sagiv & Schwartz, 2022) and support for immigration and immigration policies (Davidov et al., 2020; Davidov & Meuleman, 2012; Miglietta et al., 2018).

## The Present Study

**Figure 1:**
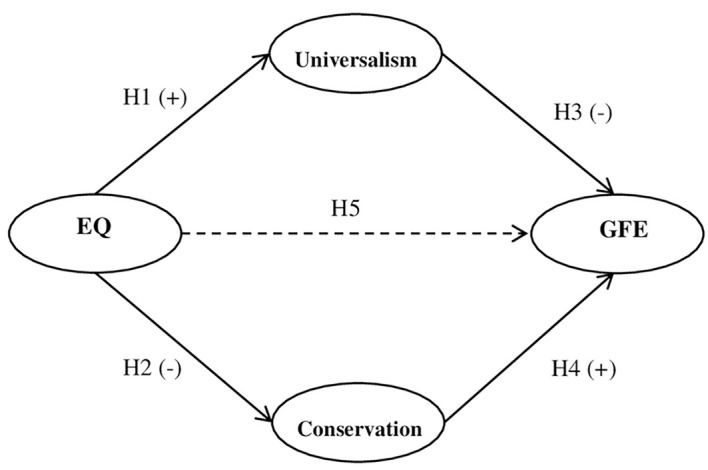
Hypothesized Model *Note.* EQ = Existential Quest; GFE = group focused enmity. Continuous lines indicate expected association. Dashed line indicates unexpected association.

Drawing on the above cited literature, we hypothesized the relationship patterns shown in Figure 1. Specifically, we analyzed whether EQ would be positively associated with the value of universalism (H1) and negatively associated with conservative values (tradition, conformity, and security) (H2); we also expected a negative association between universalism and GFE (H3) and, on the contrary, a positive association between conservative values and GFE (H4). Moreover, we expected a complete mediated relationship between EQ and GFE, that is, the direct association should no longer be significant when controlling for the mediator-outcome pathway (H5).

## Method

### Participants and Procedure

The participants were 1136 Italian adults (69.6% female) with ages ranging from 18 to 83 (*M*_age_ = 30.4; *SD*_age_. = 11.8). Nearly half of the respondents had a bachelor’s degree or higher (47.7%), while 46.8% had a high school diploma. The remaining participants (5.5%) had lower levels of education.

Respondents completed an online questionnaire in 2018 (January–June). The researchers used a snowball sampling by first contacting personal contacts via e-mail and asking them to forward the research opportunity, including the informed consent form and link to the online questionnaire, to their personal contacts. Participants filled in a set of self-report measures that on average required 20 minutes to complete through a university survey platform. Participants volunteered after giving their consent to participate; minors could not participate in the study. The dataset did not contain any missing data; the Ethics Committee of the local university approved the study protocol.

### Measures

#### Existential Quest (EQ)

The EQ scale (Van Pachterbeke et al., 2012) consists of 9 items assessing individual flexibility toward existential issues (sample items are “Today, I still wonder about the meaning and goal of my life”; “My way of seeing the world is certainly going to change again”). Participants gave their responses on a seven-point scale ranging from 1 = completely disagree to 7 = completely agree. In line with the Italian validation study (Rizzo et al., 2019), one reverse item, “I know perfectly well what the goal of my life is”, was excluded from the analyses. The Cronbach’s alpha coefficient of the 8 items was .73 in the current study, in line with the coefficient of the Italian validation (.70; Rizzo et al., 2019).

#### Personal Values (Portrait Values Questionnaire-Short)

The 21-item Portrait Values Questionnaire (Schwartz, 2003) measures the ten personal values as a verbal portrait representing the motivations, goals, and aspirations of a respondent. For each item, respondents answer the question ‘‘How much like you is this person?” by using a six-point scale ranging from 1 = not like me at all to 6 = very much like me. This study considered only the 9 items referred to the values of universalism (three items, e.g., “It is important to her to listen to people who are different from her. Even when she disagrees with them, she still wants to understand them”), security (two items, e.g., “It is important to her to live in secure surroundings. She avoids anything that might endanger her safety”), conformity (two items, e.g., “It is important to her always to behave properly. She wants to avoid doing anything people would say is wrong”) and tradition (two items, e.g., “It is important to her to be humble and modest. She tries not to draw attention to herself”). Cronbach’s alpha for the universalism was .61. Cronbach’s alpha for the conformity and security were .61 and .70, respectively; the Cronbach’s alpha for the tradition was .34. Overall, these results were in line with previous cross-country studies, including the low value of alpha coefficient relative to the tradition value items (universalism: .45; conformity: .37; security: .51; tradition: .37; Schwartz et al., 2015).

#### Group-Focused Enmity (GFE)

The GFE (Zick et al., 2008) scale measures several targets of prejudice to form an indicator of a general syndrome. According to the authors, each target of prejudice is measured by two items representing a negative attitude toward a specific outgroup. In this study, we considered targets of prejudice consistent with the Italian context. Specifically, we adapted the items from the original GFE scale (Zick et al., 2008) to cover the following six specific types of prejudice: sexism, devaluation of homosexual persons, racism, Islamophobia, devaluation of newcomers, and xenophobia. For example, sexism asks for the agreement with the following two statements: “Women should think stronger on the role as wives and mothers” and “It is more important for a wife to help her husband’s career than to have one herself”. Respondents gave their responses on a five-point scale ranging from 1 = completely disagree to 5 = completely agree.

The overall Cronbach’s alpha coefficient for these six target groups was .91, higher than the coefficient of the Italian validation study (.74; Zick et al., 2011).

#### Sociodemographic Data

A list of sociodemographic items, including respondents’ gender, age, and education, was included.

### Data Analysis

Statistical analysis was conducted using MPLUS 8 (Muthén & Muthén, 2017) and SPSS 27.0 (IBM SPSS Statistics, IBM Corporation). First, we ran confirmatory factor analyses (CFA) to examine the measurement properties of the scales in the current study. Then, a structural equation model (SEM) was estimated to test our hypotheses.

Items with 6 or more response categories (universalism, tradition, conformity, security, existential quest items) were treated as continuous variables, while items with 5 response categories were treated as ordinal variables (GFE). As pointed out by Rhemtulla and colleagues (2012) treating items with few categories as continuous variables may be a weak strategy.

Since the data did not meet the assumption of multivariate normality—as resulted from Mardia’s multivariate skewness (168.6, *p* < .001) and kurtosis (1312.0, *p* < .001) measures (Wang & Wang, 2019)—we used the Asparouhov and Muthén (2010) mean- and variance-adjusted ML method of estimation (MLMV) for continuous variables, and the mean- and variance-adjusted weighted least squares (WLSMV) for ordinal variables.

To assess the model’s goodness of fit, we applied the following criteria: root mean square error of approximation (RMSEA) ≤ .080; comparative fit index (CFI) ≥ .900; only for continuous variables standardized root mean square residual (SRMR) ≤ .080 (Browne & Cudeck, 1993; Hu & Bentler, 1999).

We used bootstrap estimation for the mediation tests (Hayes, 2018) with 5,000 samples, and we computed the bias-corrected 95% CI by determining the effects at the 2.5th and the 97.5th percentile; the indirect effects are significant when 0 was not included in the CI.

## Results

### Descriptive Statistics and Correlations

Means, standard deviations, and bivariate correlations for the variables in the study are presented in Table 1. EQ was slightly positively correlated with universalism and slightly negatively correlated with GFE. All the values included in the present study (universalism, tradition, conformity, security) were significantly correlated with GFE. In detail, universalism showed a moderate and negative correlation with GFE, while tradition, and security showed low and positive correlations with GFE.

**Table 1:** Means, Standard Deviations, and Zero-Order Correlations

Variable	M	SD	1	2	3	4	5	6
1. EQ	5.12	0.89	–					
2. Universalism	5.10	0.76	.18***	–				
3. Security	4.29	1.17	.03	.08***	–			
4. Tradition	3.55	1.04	.03	.13***	.26***	–		
5. Conformity	4.17	1.09	-.01	.16***	.33***	.38***	–	
6. GFE	1.41	0.60	-.13***	-.40***	.16***	.18***	.02	–

*Note*. *N* = 1136. EQ = existential quest. GFE = group focused enmity.

***p < .001.

### Measurement Model Testing

Confirmatory factor analyses (CFA) were conducted to test the structures of each scale. Factor loadings were freely estimated, and the latent variance was fixed at 1.0. The standardized factor loadings for the CFAs are reported in Appendix A.

The results of the EQ scale confirmed the same structure emerged in the Italian adaptation (Rizzo et al., 2019). Thus, the one-factor model fit the data satisfactorily after estimating the residual correlation of two pairs of items: χ^2^(18) = 144.0, *p* < 0.01, RMSEA = .079, 90% CI [0.67, 0.91], CFI = .926, and SRMR = .045. In line with the Italian validation study (Rizzo et al., 2019) all the factor loadings (standardized values) were acceptable except the lower factor loadings concerning the item “I often reappraise my opinion on religious/spiritual beliefs” (.27).

Regarding the factor structure of the GFE scale, the 6-factor model (Zick et al., 2008), in which each factor corresponds to one of the 6 targets of prejudice, showed extremely high correlations (from 0.95 to 1.06) between the factors concerning the following targets: xenophobia, racism, devaluation of newcomers, and Islamophobia. By examining the content of the items, all of them seem to share a common reference to the devaluation of strangers, and a new model in which all these items loaded onto a single latent factor named “devaluation of foreigners” was tested, obtaining satisfactory results. Then, following Zick and colleagues (2008), we performed a second-order CFA in which the devaluation of foreigners, devaluation of homosexuals, and sexism loaded onto a second-order factor named GFE. The second-order model fit the data very well: χ^2^(51) = 288.2, *p* < .01, RMSEA = .064, 90% CI [0.57, 0.71], CFI = .985, and the factor loadings of the three first-order factors onto the second-order factor were high, ranging from .84 to .88.

Finally, we tested the factor structure of the 9 items measuring the values of universalism, security, tradition, and conformity. We estimated a model with two correlated first-order factors concerning Universalism (3 items) and Conservation (6 items belonging to the conformity, tradition, and security subscales) (see Cieciuch & Davidov, 2012). The results showed a satisfactory model fit after estimating the residual correlation of two pairs of items of the Conservation factor: χ^2^(24) = 159.7, *p* < .01, RMSEA = .071, 90% CI [0.60, 0.81], CFI = .900, and SRMR = .054. The loading size (standardized) was acceptable, ranging from .33 to .67.

### Structural Model Testing

**Figure 2:**
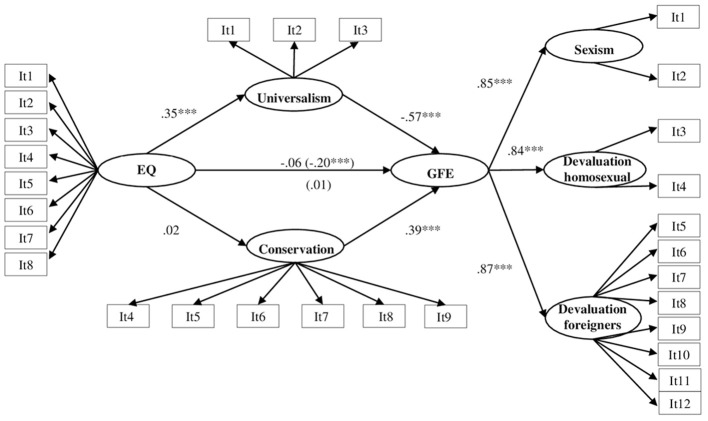
Results of the Structural Equation Model *Note.* EQ = existential quest. GFE = group-focused enmity. It = Item. Standardized values are presented. Standardized values of indicators of latent variables are omitted for the sake of clarity. Indirect effects in brackets. ***p < .001.

The standardized coefficients of the structural part of the model are depicted in Figure 2. Regarding the measurement part of the model EQ, universalism, conservation, sexism, devaluation of homosexual persons, and devaluation of foreigners were measured by their corresponding items.

The model fit the data quite well: χ^2^(366) = 1769.6, *p* < .001, RMSEA = .058, 90% CI [0.55, 0.61], CFI = .916, and explained approximately 50.5% of the variance for GFE. Table 2 shows standardized coefficients (β) and unstandardized coefficients (*B*) with their standard errors (SE) and bias-corrected 95% CI for direct, indirect, and total effects.

**Table 2:** Summary of Direct, Indirect, and Total Effects

Paths	β	*B*	*SE*	95% CI [*LL, UL*]
*Direct effects*				
EQ → Universalism	.35***	0.37***	0.07	[0.27, 0.49]
EQ → Conservation	.02	0.02	0.06	[-0.07, 0.12]
EQ → GFE	-.06	-0.09	0.06	[-0.19, 0.02]
Universalism → GFE	-.57***	-0.76***	0.09	[-0.93, -0.63]
Conservation → GFE	.39***	0.56***	0.11	[0.39, 0.76]
*Indirect effects*				
EQ → Universalism → GFE	-.20***	-0.28***	0.06	[-0.39, -0.20]
EQ → Conservation → GFE	.01	0.01	0.03	[-0.04, 0.06]
*Total effects*				
EQ → GFE	-.25***	-0.36***	0.08	[-0.49, -0.24]

*Note*. *N* = 1136. EQ = existential quest. GFE = group focused enmity. *LL* = Lower Limit. *UL* = Upper Limit.

***p < .001.

Results confirmed H1 because EQ was positively associated with the value of universalism. However, H2 was not confirmed due to an absence of relationship between EQ and conservation. We found a negative association between universalism and GFE syndrome, as expected in H3. Furthermore, H4 was also confirmed because conservation was positively related to GFE syndrome.

Finally, we confirmed H5 regarding the mediation of values between EQ and GFE. In detail, we found a negative indirect effect of universalism, but not of conservation, in the relationship between EQ and GFE. As a control analysis, we retested the same model as in Figure 2 with the inclusion of the sociodemographic variables of gender, age, and education level. The model fit was good, χ^2^(441) = 2194.6, *p* < .001, RMSEA = .059, 90% CI [0.57, 0.62], CFI = .902, and the explained variance of the outcome variable was 56.2%. The addition of these variables did not substantially change the coefficients shown in Figure 2 (see Appendix B).

## Discussion and Conclusion

The aim of the present study was to investigate the role of flexibility on existential issues in individual acceptance of social inequalities. In doing so, we have conceptualized the acceptance of inequalities as encompassing generalized prejudices and advocacy of conservation values that are at the core of ideologies of inequality. The study draws on Jost’s model of the relationship between epistemic needs and intergroup attitudes (Jost et al., 2009; Jost, Blount, et al., 2003; Jost, Glaser, et al., 2003). Specifically, we hypothesized that the propensity for existential questions influences individuals' value priorities and leads people to place more importance to the values of universalism at the expense of conservation values which are at the core of the ideology of inequality. In turn, universalism reduces generalized the prejudice against minorities, expressed in the GFE syndrome.

Although studies on EQ are still limited, the available literature suggests a promising relationship between this concept and derogatory attitudes (see Uzarevic et al, 2021). Moreover, a significant relationship has been found between EQ and the need for cognitive closure (Rizzo et al., 2019), which led us to consider EQ as a psychological variable belonging to epistemic needs (Jost, Blount, et al., 2003; Jost, Glaser, et al., 2003). Based on these assumptions, we tested direct associations of EQ with values of conservation and universalism, such as a relationship between EQ and the GFE syndrome mediated by values of conservation and universalism.

In the present work, we found a partial confirmation of our hypotheses. Since EQ was positively related to the value of universalism, we confirmed H1. This result seems to be consistent with the idea that an individual tendency to grapple with existential questions, and the related willingness to question one's belief system, may favor the endorsement of values related to the moral domain such as universalism. Indeed, the quest orientation drives people to develop empathic concern and perspective-taking, and it increases tolerance and acceptance of diversity (Arrowood et al., 2022). As defined by Schwartz (2012), universalism represents a value that emphasizes the idea of support, protection, and acceptance of all people. The ability to tolerate others even if they are not part of one's own group, as supported by the adjacency of values of universalism and benevolence in Schwartz’s (1992) circumplex model seems to be consistent with the individual tendency to be open to other people and possibilities as conceptualized by EQ. As for H2, we found a lack of significant relationship between EQ and conservative values (security, tradition, and conformity) in place of the expected negative one. This result suggests that individual efforts to ask existential questions do not challenge values outside the moral domain, as individual striving for security and the subordination of the self to the socially imposed expectations (Schwartz, 2012). Moreover, as noted earlier, previous studies have confirmed a conceptual difference between a general cognitive style and EQ (Rizzo et al., 2019) that may partially explain the lack of a specific link between EQ and conservation. The relationships between values and prejudicial attitudes were confirmed (H3 and H4) in accordance with the vast literature (Beierlein et al., 2016; Davidov et al., 2020; Miglietta et al., 2018; Souchon et al., 2017). According to H5, we found mediation of values in the relationship between EQ and GFE syndrome. Specifically, the total effect of EQ on GFE syndrome was due to mediation of universalism (but not conservation), whereas no direct relationship emerged between EQ and GFE syndrome. In other words, our results suggest on the one hand that EQ promotes values of open-mindedness, social justice, and equality. This contributes to the reduction of generalized prejudice against minorities likely reducing individuals’ acceptance of social inequalities. On the other hand, however, EQ does not help reduce the acceptance of conservation values that are at the core of the ideology of inequality. This finding represents the most important contribution of the current work to the literature, as it clarifies the role of EQ in avoiding prejudicial attitudes.

In summary, this study offers insights into explaining EQ as one of the drivers that lead people to support certain values by tending to accept other perspectives. In particular, the study highlights the importance of the moral dimension in forming attitudes toward devalued minorities. This is consistent with previous studies that emphasise the particular nature of EQ in relation to a general need for cognition (Rizzo et al., 2019; Van Pachterbeke et al., 2012), as well as the role that EQ can play in addressing moral dilemmas and cultural/religious issues. For example, EQ has been shown to play an important role in the acceptance of moral dilemmas such as abortion, child euthanasia, homosexual adoption, and suicide (Deak & Saroglou, 2015). In addition, EQ has been found to be important in the successful acculturation process of second-generation immigrants who belong to a religion other than the majority religion in European countries (Rizzo et al., 2022). More broadly, the present study helps to clarify that the relationship found between EQ and GFE is not just a special case of the general relationship between the need for closure and tolerance or prejudice. To the best of our knowledge, no other study has examined the relationship between willingness to think about existential issues and personal values (Schwartz, 2012). Moreover, this finding indicates that consideration should be given to including EQ as a psychological variable in epistemic needs, as is the case with similar constructs related to general close-mindedness (Jost et al., 2009; Jost, Blount, et al., 2003; Jost, Glaser, et al., 2003). Future studies are needed to better explain the role of EQ in value endorsement and its role within epistemic needs. Following Jost and colleagues (2009), it might be interesting, for example, to understand the role of EQ as an epistemic need in advocating a political ideology. Furthermore, experimental designs can help to understand whether levels of EQ vary with respect to the importance of existential concerns and how the variations affect the moral dimension that defines orientation to the existential quest (Arrowood et al., 2022). Moreover, future research should take into account the role of different cultures and religions across countries in the study of EQ, values, and prejudice. Indeed, in Western societies characterized by the presence of different cultures, religions, and/or sexual orientations, a willingness to reflect on the meaning of life and to accept doubt and/or different perspectives may help people understand the various facets of our contemporary society (see Rizzo et al., 2022).

The current study is not exempt from limitations. Although this study represents a first attempt to examine the influence of EQ on prejudice, a limitation is its cross-sectional nature which makes it impossible to establish causal relationships among the variables in the study. Future longitudinal studies could confirm the effective influence of EQ on prejudicial attitudes and its relationship with personal values (Schwartz, 2012). Another limitation concerns the generalizability of our results. Our participants were recruited in only one European country and only in one part of the country (northwestern Italy). In addition, the snowball sampling method, which began with university students, resulted in a partially unbalanced sample in terms of gender (most participants were women), educational level (the majority had at least a secondary school certificate), and age (mainly young adults). Concerning the limitations on the measures used in the present study, we found low reliability for the items related to tradition value. As has been noted in other work (e.g., Schwartz et al., 2015), the tradition value tends to show low reliability. However, for the main results of the study, we used a latent variable of conservation that included all items related to tradition, conformity, and security values, all of which showed acceptable loading on the measure of the construct of interest (see Appendix A). Nevertheless, this study suggests some important practical implications. Indeed, being flexible on existential issues may help people develop greater acceptance of minorities by reducing generalized prejudice. Interventions aimed at promoting social inclusion could work at the cognitive level and improve individuals' ability to think critically about existential issues. The role of individual reflection on existential issues in activating universalism suggests further implications, particularly for educational programs aimed at preventing the stigmatization of minority groups in the future. In this respect, Galamba and Matthews (2021) emphasized that to combat racism, sexism, and other prejudice against minority groups, it is important to strengthen mutual respect and the idea of the common good among students, pointing to educational settings as the primary place where social values and shared norms are learned.

## Figures and Tables

**Appendix B:**
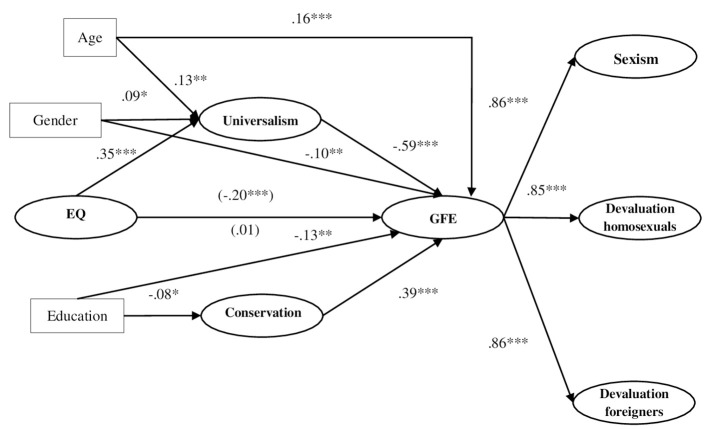
Standardized Results for the Structural Model With Age, Education, and Gender as Control Variables *Note.* EQ = existential quest. GFE = group-focused enmity. Standardized values are presented. Indicators of latent variables are omitted for the sake of clarity. Only significant standardized values and arrows are reported for the sake of clarity. Indirect effects are in brackets (bootstrap 5000). Gender: 0 = Male; Education was recoded as a dummy variable: 0 = low-middle education.

## Appendices

**Table:** Appendix A: Standardized Loadings for CFAs

EQ	Item	Loading
	1. Today, I still wonder about the meaning and goal of my life.	.31
	2. My attitude toward religion/spirituality is likely to change according to my life experiences.	.33
	3. Being able to doubt about one’s convictions and to reappraise them is a good quality.	.52
	4. In my opinion, doubt is important in existential questions.	.48
	5. My way of seeing the world is certainly going to change again.	.82
	6. My opinion varies on a lot of subjects.	.71
	7. Years go by, but my way of seeing the world doesn’t change.	.50
	8. I often reappraise my opinion on religious/spiritual beliefs.	.27
PVQ-short	Item	Loading
Universalism	1. He/ She thinks it is important that every person in the world should be treated equally. He/ She believes everyone should have equal opportunities in life.	.67
	2. It is important to him/ her to listen to people who are different from him/ her. Even when he/ she disagrees with them, he/ she still wants to understand them.	.59
	3. He/ She strongly believes that people should care for nature. Looking after the environment is important to him/her.	.53
Conservation	4. He/ She believes that people should do what they’re told. He/ She thinks people should follow rules at all times, even when no one is watching.	.64
	5. It is important to him always to behave properly. He/ She wants to avoid doing anything people would say is wrong.	.67
	6. It is important to him/ her to be humble and modest. He/ She tries not to draw attention to himself/ herself.	.45
	7. Tradition is important to him/ her. He/ She tries to follow the customs handed down by his/ her religion or his/ her family.	.42
	8. It is important to him/ her to live in secure surroundings. He/ She avoids anything that might endanger his/ her safety.	.43
	9. It is important to him/ her that the government ensures his/ her safety against all threats. He/ She wants the state to be strong so it can defend its citizens.	.33
GFE	Item	Loading
Sexism	1. Women should think stronger on the role as wives and mothers.	.79
	2. It is more important for a wife to help her husband’s career than to have one herself	.89
Devaluation homosexuals	3. It is disgusting when homosexuals kiss in public.	.88
	4. Marriages between two women or between two men should be permitted.	.89
Devaluation foreigners	5. There are too many foreigners living in Italy.	.89
	6. When jobs get scarce, the foreigners living in Italy should be sent (back) home.	.89
	7. Those who are new somewhere should be content with less.	.81
	8. Those who have always been living here should have more rights than those who came later.	.88
	9. Italian re-settlers should be better off than foreigners because they are of Italian origin.	.91
	10. It is right that Whites are leading in the world.	.77
	11. Immigration to Italy should be forbidden for Muslims.	.83
	12. With so many Muslims in Italy, one feels increasingly like a stranger in one’s own country.	.88

*Note*. *N* = 1136. EQ = existential quest. PVQ-short = Portrait Values Questionnaire-short. GFE = group focused enmity.
